# Peripheral blood marker of residual acute leukemia after hematopoietic cell transplantation using multi-plex digital droplet PCR

**DOI:** 10.3389/fimmu.2022.999298

**Published:** 2022-09-29

**Authors:** M. Stanojevic, M. Grant, S. K. Vesely, S. Knoblach, C. G. Kanakry, J. Nazarian, E. Panditharatna, K. Panchapakesan, R. E. Gress, J. Holter-Chakrabarty, Kirsten M. Williams

**Affiliations:** ^1^ Department of Pediatrics, MedStar Georgetown University Hospital, Washington, DC, United States; ^2^ Aflac Cancer and Blood Disorders Center, Children’s Healthcare of Atlanta, Emory University, Atlanta, GA, United States; ^3^ Stephenson Cancer Center, University of Oklahoma Health Sciences Center, Oklahoma, OK, United States; ^4^ Children’s Research Institute, Research Center for Genetic Medicine, Children’s National Health System, Washington, DC, United States; ^5^ Experimental Transplantation and Immunotherapy Branch, National Cancer Institute, National Institutes of Health, Bethesda, MD, United States; ^6^ Department of Oncology, Children’s Research Center, University Children’s Hospital Zurich, Zurich, Switzerland; ^7^ Department of Pediatric Oncology, Dana-Farber Boston Children’s Cancer and Blood Disorders Center, Boston, MA, United States

**Keywords:** minimal residual disease, digital droplet PCR, acute leukemia, relapse, hematopoietic cell transplantation

## Abstract

**Background:**

Relapse remains the primary cause of death after hematopoietic cell transplantation (HCT) for acute leukemia. The ability to identify minimal/measurable residual disease (MRD) *via* the blood could identify patients earlier when immunologic interventions may be more successful. We evaluated a new test that could quantify blood tumor mRNA as leukemia MRD surveillance using droplet digital PCR (ddPCR).

**Methods:**

The multiplex ddPCR assay was developed using tumor cell lines positive for the tumor associated antigens (TAA: WT1, PRAME, BIRC5), with homeostatic ABL1. On IRB-approved protocols, RNA was isolated from mononuclear cells from acute leukemia patients after HCT (n = 31 subjects; n = 91 specimens) and healthy donors (n = 20). ddPCR simultaneously quantitated mRNA expression of WT1, PRAME, BIRC5, and ABL1 and the TAA/ABL1 blood ratio was measured in patients with and without active leukemia after HCT.

**Results:**

Tumor cell lines confirmed quantitation of TAAs. In patients with active acute leukemia after HCT (MRD+ or relapse; n=19), the blood levels of WT1/ABL1, PRAME/ABL1, and BIRC5/ABL1 exceeded healthy donors (p<0.0001, p=0.0286, and p=0.0064 respectively). Active disease status was associated with TAA positivity (1+ TAA vs 0 TAA) with an odds ratio=10.67, (p=0.0070, 95% confidence interval 1.91 – 59.62). The area under the curve is 0.7544. Changes in ddPCR correlated with disease response captured on standard of care tests, accurately denoting positive or negative disease burden in 15/16 (95%). Of patients with MRD+ or relapsed leukemia after HCT, 84% were positive for at least one TAA/ABL1 in the peripheral blood. In summary, we have developed a new method for blood MRD monitoring of leukemia after HCT and present preliminary data that the TAA/ABL1 ratio may may serve as a novel surrogate biomarker for relapse of acute leukemia after HCT.

## Introduction

Relapse remains the primary cause of death after hematopoietic cell transplantation (HCT) for acute leukemia (1, 2). Identification of leukemia at minute disease burden, termed minimal/measurable residual disease (MRD), could potentially improve outcomes, permitting intervention when the disease burden is low, when these interventions are most likely to be successful (3). The presence of MRD has been validated as a biomarker of disease response, recurrence, and prognosis in acute leukemia (3–11). Technologic improvements in evaluation of disease burden have continued to redefine this MRD threshold of measurable disease, from 10^-2^ based on morphology, to currently 10^-4^ using flow cytometry, and to 10^-5^ with real-time quantitate polymerase chain reaction (qPCR) for known molecular targets, or even up to 10^-6^ using next generation sequencing to detect the immunoglobulin receptor of B-cell acute lymphoblastic leukemia in the marrow, or to identify sensitive error-corrected MRD to detect AML (8, 12–15). This increased sensitivity to detect MRD has enhanced prediction of relapse and improved disease stratification to allocate intensive therapies to those with persistent disease, sparing these toxicities for chemotherapy-sensitive cancers (13). However, many leukemias, and specifically up to 70% of acute myeloid leukemias, lack targets for these more sensitive modalities and most of these tests require invasive bone marrow tests, limiting the frequency of monitoring (13).

In contrast, levels of tumor nucleic acids detected in peripheral blood permit more frequent monitoring of MRD, which increases the likelihood of early detection of MRD. The ability to quickly monitor disease using the BCR/ABL1 fusion ratio in ALL is an example of such monitoring. As targets such as this fusion are uncommon in acute leukemias, measurement of common tumor-associated antigen cell-free nucleic acid could afford a more broadly applicable MRD test for post HCT monitoring.

Digital droplet PCR (ddPCR) can sensitively quantify the target messenger RNA (mRNA) at a low level of disease burden using a small number of cells. The ddPCR technology partitions the sample into many individual PCR reactions, permitting lower limits of detection for measurement of candidate targets. Partitions are resulted as positive or negative per individual reaction, based on fluorescence signal emitted. This method allows absolute quantification of target DNA molecules without reference standard curves (16). Studies have shown that ddPCR has high sensitivity and precision even when quantifying low-abundance targets (17, 18). In addition, ddPCR can simultaneously measure up to four targets in a single experiment, increasing the likelihood of capturing a target candidate antigen (19). This permits quantification of three tumor-associated targets and a single homeostatic reference gene, with the ratio of these providing standardization to measure disease burden over time. ABL1 has been recommended as the best reference gene for MRD detection (20). To date, ddPCR has been used to detect MRD in AML *via* testing for NPM1 mutation, DNMT3A, IDH1/2, and BCR-ABL fusion, relatively uncommon targets in acute leukemia (21–25). A novel approach of double drop-off digital droplet PCR has been used to measure gene mutations in NPM1, IGH2, and NRAS, enabling sensitive monitoring from the peripheral blood in AMLs with these mutations (26). DdPCR has also been used to measure WT1 expression in patients with AML, demonstrating good concordance with qPCR measurements with high accuracy and precision (27, 28). Another study demonstrated a correlation between ddPCR-determined WT1 copy numbers and CD123+ cells detected by flow cytometry in AML patients, and linked higher WT1 levels to poorer survival (29). ddPCR has consistently demonstrated high precision, accuracy, and the ability to detect very low levels of DNA or RNA copies from few cells, supporting the hypothesis that it may be valuable for monitoring leukemia relapse after HCT (30).

We hypothesized that a blood-based multiplex ddPCR assay of WT1, BIRC5 (survivin), and PRAME as a ratio against homeostatic ABL1 would detect MRD in the majority of relapsed refractory acute leukemias after HCT, at levels exceeding those of healthy individuals. The candidate targets of Wilms tumor 1 (WT1), survivin (BIRC5), and PRAME were selected given the high prevalence in relapsed and refractory acute leukemias (77-81% for WT1, 63-100% for BIRC5, and 42-87% for PRAME) (31–38). These tumor associated antigens are poorly expressed in healthy tissues, including podocytes of the kidney (WT1), hematopoietic stem cells (WT1, survivin), and fat and gonadal cells (survivin), though are present in fetuses (39–41). WT1 and PRAME have been shown to identify AML MRD by qPCR, and have been linked to prognosis (42). Blood levels of WT1 have been shown to correlate with marrow disease burden by qPCR and have been reported to sensitively detect relapse prior to standard of care tests (42). However, the quantity of cells required for qPCR limits the ability to use this method to test >1 target, especially in pediatric patients (43–52). To date, WT1, BIRC5, and PRAME have not been promoted as biomarkers of relapse, likely in part due to the scant data for BIRC5 and PRAME and the lack of sensitivity for detection of relapse in prior studies of WT1 in acute leukemia (3, 53).

Here, we present data showing that this assay simultaneously detected and quantified mRNA levels of three tumor associated antigens (TAAs) as a ratio to a homeostatic ABL1 in the peripheral blood and preliminary data of ability of this test to detect MRD in relapsed acute leukemia after HCT in the peripheral blood.

## Materials and methods

### Patient samples

Samples were obtained from patients who had undergone HCT for acute leukemia enrolled on IRB-approved protocols. All samples were deidentified prior to analysis; the laboratory technicians were blinded as to disease status at the time of sampling. MRD+ was defined as detectable leukemia by standard of care tests including flow cytometry, fluorescence *in situ* hybridization, or polymerase chain reactions but undetectable by conventional cytomorphologic criteria. Those who were MRD negative could not be detected by any of these methods. ≥MRD+ was used to characterize patients that had disease detected by either conventional cytomorphologic criteria and/or by the other MRD standard of care tests.

### PBMC isolation

5-10 mL of whole blood was collected from patients and healthy donors in sodium-heparin tubes and maintained on a rocker at room temperature until processing. Peripheral blood mononuclear cells (PBMCs) were isolated from whole blood and marrow by density gradient centrifugation using SepMate™ tubes (Stemcell Technologies, Vancouver, BC, Canada) and Lymphocyte Separation Medium (MP Biomedicals, Solon, Ohio, USA) according to the manufacturer’s protocol. PBMCs were cryopreserved and stored in liquid nitrogen vapor phase until further use.

### Tumor cell culture

The K562 (Chronic myelogenous leukemia) and Hep3B (Hepatocellular carcinoma) tumor cell lines were obtained from ATCC (Manassas, VA, USA) and used as positive tumor controls, as K562 express WT1 and PRAME and Hep3B expresses BIRC5. K562 cells were cultured in Iscove’s Modified Dulbecco’s Medium (ATCC; Cat No. 30-2005) containing 10% FBS in a 5% CO_2_ incubator at 37°C. Hep3B cells were cultured in Eagle’s Minimum Essential Medium (ATCC; Cat No. 30-2003) containing 10% FBS in a 5% CO_2_ incubator at 37°C.

### RNA isolation

Total RNA was extracted from 3-5 million mononuclear cells of healthy subjects, leukemia patients, and tumor cell lines using RNeasy Mini Kit following the manufacturers’ protocol (Qiagen, Valencia, CA, USA). An on-column DNase digestion was performed using the RNase-free DNase kit (Qiagen, Valencia, CA, USA). The RNA concentration and purity were assessed using a NanoDrop spectrophotometer (NanoDrop Technologies, Wilmington, DE, USA). RNA quality was further determined using the Agilent 2100 Bioanalyzer (Agilent Technologies, Santa Clara, CA, USA). RNA was determined to be of sufficient quality if the RNA integrity number (RIN) exceeded 8. RNA was stored at -80°C until further use.

### Complementary DNA synthesis

Complementary DNA (cDNA) was synthesized by reverse-transcription from 1 µg of total RNA using High-Capacity cDNA Reverse Transcription Kit (Thermo Fisher Scientific, Waltham, MA, USA), and stored at -20°C until use.

### Primers and probes design

Initially, predesigned TaqMan assays were used in order for simultaneously detection of TAAs of interest. TaqMan assays demonstrated our targets but showed nonspecific annealing and poor cluster separation. To improve probe specificity and separation of clusters, primers and probes were custom designed to conserved regions to simultaneously detect all transcript variants for each gene (Integrated DNA Technologies, Coralville, IA, USA). We selected ABL1 as a control gene, due to consistent levels in healthy and tumor cells (20).

### Digital droplet PCR

Droplet digital PCR (ddPCR) experiments were performed and analyzed according to the RainDrop digital PCR system (RainDance Technologies, Billerica, MA, USA). The ddPCR reaction mixture contained 1X TaqMan genotyping master mix (Life technologies, Carlsbad, CA, USA) as recommended in the Raindance protocol; 1X RainDance droplet stabilizer; primers and probes and 150 ng of template cDNA in a reaction volume of 50µL. To mimic patient samples, 120 ng of cDNA from healthy donor peripheral blood mononuclear cells (PBMC) was spiked with 15 ng of cDNA from each tumor cell line. Droplets were generated using the RainDrop Source instrument. Each sample was partitioned into approximately 10 million, 5 pL droplets. Thermal cycling was performed using the Tetrad 2 Peltier thermal cycler following manufacturer’s recommendations to amplify the targets. (BioRad, San Diego, CA, USA). The samples were transferred to a RainDrop Sense instrument for capture of fluorescence intensity of each droplet which determines droplet positivity. Data were analyzed using the RainDrop Analyst software. Spectral compensation was applied to all samples using a calculated matrix. The gates for the identified clusters were manually selected based on no template and positive controls, and single-plex assays for each target. The sample with the highest transcript count was used as a control to define the gates. To develop the multiplex assay, single plots were first performed and the probe concentrations altered to optimize the separation between the 4 targets, WT1, PRAME, BIRC5, and ABL1, as performed previously by our group (54). The levels of WT1, PRAME, and BIRC5 were normalized to the levels of ABL1. For patient samples, each run included a no template control (sterilized, RNAse, DNAse free, water) and a positive control (healthy donor cells spiked with K562 and Hep3B cells). All experiments were performed in triplicate, and the mean value used to generate the TAA/ABL1 ratios. These methods and replicates were designed in accordance with published recommendations for ddPCR (55).

### Statistical methods

Descriptive statistics were calculated. The distribution of ABL ratios were compared between healthy donors and patients with active leukemia using the Wilcoxon two-sample test with the t approximation. Logistic regression was performed to predict active disease status among patients using TAA positivity (1+ TAA versus 0 TAA) and to produce a receiver operator curve (ROC) to calculate area under the curve. Kaplan-Meier curves with log-rank tests were calculated to assess the association between mortality and number of positive TAA/ABL1 ratios among patients with active leukemia. A significance level of alpha equal to 0.05 was used. Analyses were conducted using SAS statistical software, version 9.4 (SAS Institute, Cary, NC).

## Results

### Multiplex ddPCR simultaneously detected ABL1, PRAME, BIRC5 and WT1

**Figure 1:**
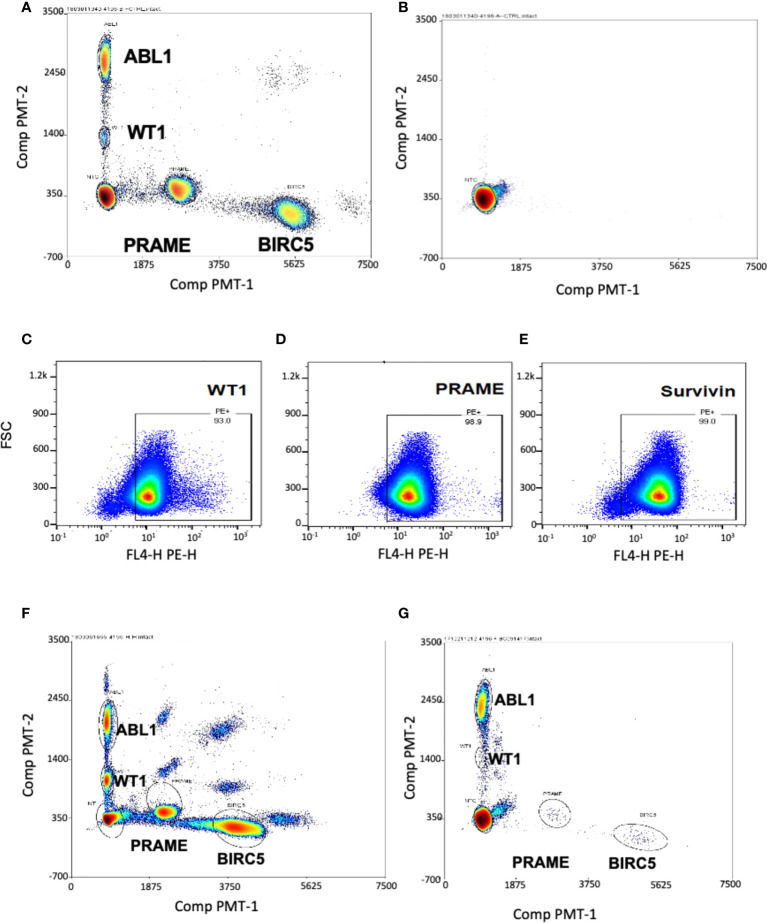
Multiplex ddPCR simultaneously detected ABL1, PRAME, BIRC5 and WT1 in cell lines and at low levels in healthy donors. Separation of single-positive droplet clusters in dual color, four-target ddPCR concomitantly detected ABL1, BIRC5, PRAME and WT1. **(A)** Healthy control PBMCs were spiked with HEP3B (BIRC5+) and K562 (WT1+, PRAME+) tumor cell lines. Multiplex ddPCR simultaneously detected and discriminated the three TAA and the housekeeping gene ABL1. **(B)** Representative dot plot showing no transcript amplification in the No Template Control. WT1 **(C)**, PRAME **(D)** and survivin **(E)** proteins were detected in the THP-1 leukemia cell line by Flow Cytometry. **(F)** All three TAA transcripts were detected in the THP-1 leukemia cell line by ddPCR. The appearance of double positive reflects artifact from the high mRNA copy numbers in the undiluted tumor which exceed the optimal threshold. **(G)** Minimal levels of the three transcripts were detected in peripheral blood of healthy donors.

To determine if these tumor antigens could be simultaneously detected and discriminated for quantitation, we used cancer cell lines known to express the three TAA: hepatoblastoma (HEP3B), which expresses BIRC5; and K562, an immortalized leukemia line that expresses WT1 and PRAME. As the goal was to optimize this test for low level blood detection of marrow leukemia, multiplex ddPCR was performed on healthy donor PBMCs spiked with HEP3B and K-562 tumor cell lines. The test simultaneously detected and discriminated homeostatic ABL1 and WT1 (both FAM fluorescent), along with PRAME and BIRC5 (both TET fluorescent) in a single assay from minimal input (2-3 million cells) (**Figure 1A** **)** (20). These cells were tested in septuplets in two separate experiments with separate healthy donors (**Supplemental Table 1**) to generate the coefficient of variation (CV), as recommended for ddPCR experiments, and then used as the positive control in all subsequent experiments (55). The TAA/ABL1 mRNA ratios in the tumor cell line-spiked samples were highly reproducible, with a CV of 0.05 and 0.11 for WT1/ABL1, 0.1 and 0.21 for PRAME/ABL1, and 0.12 and 0.21 for BIRC5/ABL1 (**Table 1** **).**

**Table 1:** Descriptive statistics of ddPCR precision in positive controls.

		WT1/ABL1	PRAME/ABL1	BIRC5/ABL1
sample1	std dev	0.0010	0.193255	0.091963
sample1	mean	0.019429	1.851429	0.742857
sample1	CV	0.05023	0.104382	0.123796
sample1	median	0.02	1.88	0.79
				
		WT1/ABL1	PRAME/ABL1	BIRC5/ABL1
sample2	std dev	0.00189	0.307013	0.066583
sample2	mean	0.017286	1.462857	0.64
sample2	CV	0.109329	0.209872	0.104036
Sample 2	median	0.016	1.31	0.61

The negative control (no template control) of sterilized, RNAse- DNAse-free water, was also re-tested in all subsequent tests, and showed no transcript amplification (**Figure 1B** **)**. As it was not possible to obtain leukemia cells from many patients to confirm that TAA detection reflected tumor expression in the patient, an acute leukemia cell line was chosen to use as a surrogate. THP-1 is a cell line for which, like our patients, expression of the three TAA was unknown. Therefore, we first determined expression of all three TAAs in THP-1 by flow cytometry (**Figures 1C-E**), and this was confirmed by the novel triplex ddPCR (**Figure 1F**).

### Healthy subjects TAA/ABL1 levels

To determine a threshold for TAA/ABL1 from peripheral blood mononuclear cells, twenty healthy donors were tested. As expected, multiplex ddPCR detected low mRNA levels for WT1, PRAME and BIRC5 (**Figure 1G**; **Supplementary Table 2**). All but one healthy donor fell within the normal limits of variability. The single outlier, a female, expressed high WT1/ABL1 (0.036), PRAME/ABL1 (1.675) and BIRC5/ABL1 (0.159) ratios. This outlier had TAA/ABL1 ratios that far exceeded those previously published for WT1/ABL1 and PRAME/ABL1 in healthy donors, similar in the remaining 19, for WT1/ABL1 (0.003 by ddPCR; 0.005 qPCR, outlier: 0.036) and PRAME/ABL1 (0.002 by ddPCR; 0.001 by qPCR; outlier 1.675) (46, 56). Because our goal was to choose the upper limit of the normal values as a strict determination of the threshold analysis, we excluded this one outlier and chose the next highest values for each of the TAAs from the 19 remaining normal donors. This resulted in upper thresholds of 0.031, 0.048 and 0.26 for WT1/ABL1, PRAME/ABL1 and BIRC5/ABL1 respectively (**Supplementary Table 2**). As the ddPCR test is designed to be serially evaluated in post-HCT recipients, with each test compared to prior levels and level at the time of disease burden pre-HCT, the importance of designating a normal threshold was to permit analysis to correlate with standard of care testing.

### Multiplex ddPCR detected TAA/ABL1 ratios in the blood of adult and pediatric patients of diverse acute leukemia subtypes after HCT

**Figure 2:**
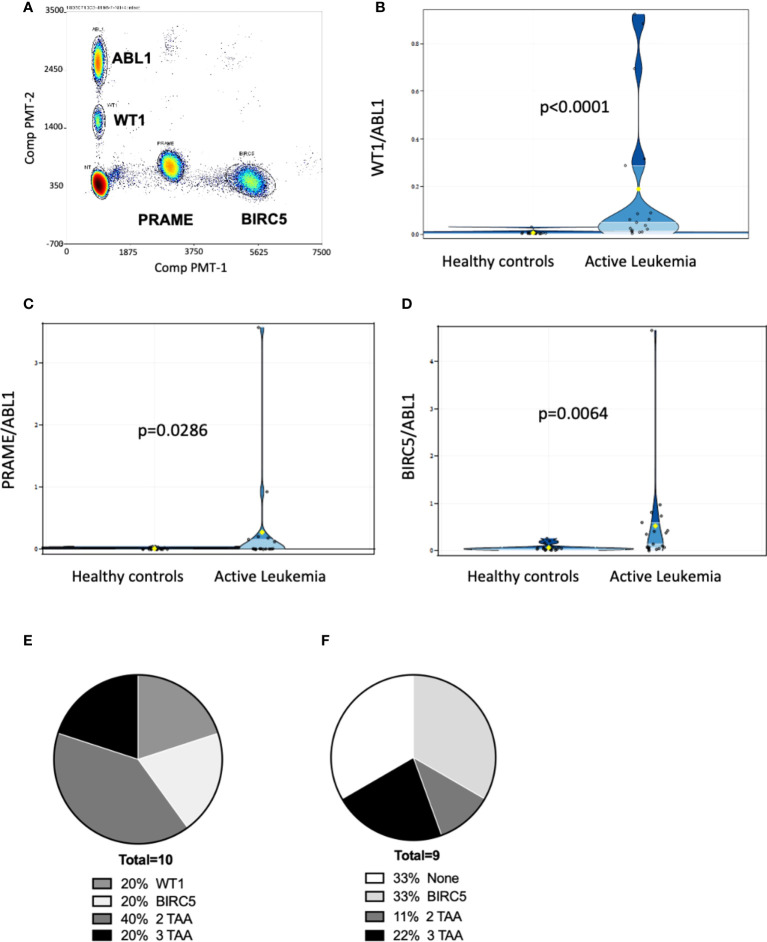
Multiplex ddPCR derived TAA/ABL1 ratios in adult and pediatric patients of diverse acute leukemia subtypes. Discrete clusters of ABL1-, and/or BIRC5-, and/or PRAME- and/or WT1-positive droplets were observed in MRD+ leukemia samples. **(A)** Representative bivariate dot plot from patient with progressive relapsed refractory leukemia, who was positive for all 3 TAA. Summary data in violin plots of WT1/ABL1 **(B)**, PRAME/ABL1 **(C)** and BIRC5/ABL1 **(D)** ratios in healthy donors (n=19) and patients who had active leukemia (MRD+: minimal residual disease or greater (relapse) by standard of care test (n=19). Where multiple timepoints existed the highest TAA/ABL1 value chosen. The mean, lower quartile, upper quartile were: 0.0976, 0.004, 0.0499 for WT1, 0.1437, 0.0019, 0.0366 for PRAME, 0.296, 0.0362, and 0.2627 for BIRC5. Summary data of TAA/ABL1 positivity for active AML [**(E)**; n = 10] and ALL [**(F)**; n = 9] in peripheral blood using the timepoint with greatest number of TAA/ABL1 if there was a change over time. TAA: tumor-associated antigen. Statistics = Wilcoxon two-sample test, t approximation.

We next determined whether ddPCR could detect blood TAA in patients who had evidence of residual acute leukemia after HCT by standard of care tests (marrow flow cytometry, BCR-ABL testing, or cytogenetic abnormalities). RNA was isolated from PBMCs, and marrow (where available) collected at various time points from 31 pediatric (n=6) and adult (n=25) patients who underwent HCT to treat acute leukemia (18 AML, 13 ALL [B-cell: 12; 1 T-cell: 1]). The median age of the cohort was 31 years, median time post-HCT 6 months. Of the 31 patients, 55% identified as Caucasian Hispanic, 10% African American, 29% Caucasian (not Hispanic) and 6% Asian. The cohort included patients with active leukemia (in relapse or minimal/measurable residual disease positive by standard of care clinical tests) and those in remission with no clinical evidence of disease (NED)) (**Table 2** **)**. Discrete clusters of ABL1-, and/or BIRC5-, and/or PRAME- and/or WT1-positive droplets were observed in active leukemia samples (**Figure 2A** **).** Of all patients tested, 61% (19/31) had evidence of active leukemia (MRD+ or overt relapse) at the time of ddPCR testing. Patients had 1 to 15 samples analyzed for the TAAs. TAA/ABL1 ratios exceeded the healthy threshold in 84% (16/19) of these patients with active leukemia after HCT. Of the 19 patients with active disease, including those leukemias without elevated TAA/ABL1, the blood levels of WT1/ABL1, PRAME/ABL1, and BIRC5/ABL1 exceeded healthy donors (p<0.0001, p=0.0286, and p=0.0064 respectively, **Figures 2B-D**).

**Table 2:** HCT Patient and Disease Characteristics and Disease Burden.

AML/ALL ID	Age (year)/M/F	Ethnicity	Disease and cytogenetic markers	Time post HCT of 1^st^ ddpCR test	≥MRD+ at ddPCR test	SOC molecular marker (BM/PB)	+ by ddPCR at any point
AML-1	39 F	African American	AML 50XX, inv (9)(p12q13), +6, +13, +13, +14	9 months	No (+ later timepoint) without relapse	None	PRAME
AML-2	26 M	Asian	Bilineage AL (T/AML)	6 months	NED	None	None
AML-3	20 M	Caucasian/Hispanic	AML	1.5 months	NED	None	None
AML-4	31 F	Caucasian/Hispanic	Secondary AML (breast) inv16	3 months	NED (+ breast CA later timepoint)	Inv16 (BM)	PRAME/BIRC5
AML-5	45 F	Caucasian/Hispanic	CML, MDS/AML 9;22 mono7, trisomy8	3 months	MRD+ BCR-ABL 0.05%, BM flow -➔ relapse 6 months	BCR-ABL (PB)	BIRC5
AML-6	41 F	Caucasian/Hispanic	CML/AML 9;22 (q34;q11.2)	1 month	Relapse BM 70-80% blasts (flow)	BCR-ABL (PB)	WT1/BIRC5/PRAME
AML-7	39 F	Caucasian/Hispanic	MDS/AML t(13;21)(q32;q22)[3] der(13)t(13;21)(q32;q22), t(8;13;13;21)(q13;q14; q32;q22; nuc ish(RUNX1T1, RUNX1)	3 months	Relapse PB blasts morph	Runx1 (BM)	WT1
AML-8	64 M	Caucasian	MDS/AML	11 months	MRD+ BM 2% blasts (flow)	None	BIRC5
AML-9	54 M	Caucasian	MDS/AML trisomy 8, 13, 20, MLL RUNX1T1	6 months	Relapse “replaced” blasts (morph)	MLL/Runx1 (BM)	WT1/BIRC5
AML-10	68 F	Caucasian	MDS/AML trisomy 8, 5q-, mono7	7 months	Relapse PB blasts morph	None	WT1/PRAME/BIRC5
AML-11	70 M	Caucasian	MDS/AML trisomy 8, 14	9 months	Relapse PB blasts morph	None	WT1/BIRC5
AML-12	58 M	Asian	AML 9q-, trisomy 21, MutCEBPa, Kit IK2F	15 months	NED by PB but low platelets (relapse + 10 weeks)	None	BIRC5
AML-13	21 M	Caucasian/Hispanic	AML GATA2	6 months	NED	None	WT1/BIRC5
AML-14	10 F	Caucasian	AML MLL NPM1	6 months	NED ➔ +relapse BM morph (4 months)	MLL (BM)	None
AML-15	4 F	Caucasian	AML MLL	15 months	MRD+ 0.01% MLL PCR	MLL (BM)	WT1
AML-16	30 M	Caucasian	AML MLL t11:16, t7:11 der7 partial trisomy 11q, ATM and Wt1 mutation	11 months	NED	MLL (BM)	None
AML-17	31 F	Caucasian/Hispanic	AML FLT3 ITD, double mutation WT1 and DNMT3a, FLT3 mutation	3 months	Relapse PB blasts morph	FLT3 (BM)	WT1/PRAME
AML-18	3 M	African American	AML complex cytogenetics + 13;14 robertsonian trans.	3 months	Relapse PB blasts morph	None	WT1/PRAME
ALL-1	19 M	Caucasian/Hispanic	Biphenotypic AL, 9:22	9 months	NED	BCR-ABL (PB)	None
ALL-2	23 M	Caucasian/Hispanic	B-ALL	0.23 months	MRD+ BM blasts 2% flow	None	BIRC5
ALL-3	22 M	Caucasian/Hispanic	B-ALL Ph+	2 months	Relapse PB blasts morph	BCR-ABL (PB)	WT1/PRAME/BIRC5/
ALL-4	27 F	Caucasian/Hispanic	B-ALL Ph+	3 months	Relapse PB blasts morph	BCR-ABL (PB)	None
ALL-5	34 F	Caucasian/Hispanic	B ALL	24 months	NED relapse 2 months later (BM morph) no ddPCR test	None	None
ALL-6	36 M	Caucasian/Hispanic	B-ALL Ph+	3 months	NED	BCR-ABL (PB)	None
ALL-7	36 F	Caucasian/Hispanic	B-ALL Ph+	6 months	Relapse PB blasts by flow	BCR-ABL (PB)	None
ALL-8	47 F	Caucasian/Hispanic	B-ALL	3 months	Relapse chloroma	None	BIRC5
ALL-9	40 M	Caucasian/Hispanic	B-ALL Ph+	9 months	NED	BCR-ABL (PB)	WT1
ALL-10	25 M	Caucasian/Hispanic	B-ALL	3 months	MRD+ BM 1% by flow	None	None
ALL-11	9 F	Caucasian	B-ALL Ph+ Li-Fraumeni	8 months	MRD+ BCR-ABL PB 0.00036	BCR-ABL (PB)	BIRC5
ALL-12	8 M	African American	B-ALL Ph	12 months	Relapse	None	WT1/PRAME/BIRC5
ALL-13	7 M	Caucasian	T-ALL	2 months	Relapse Testicular mass, BM 20% blasts flow, PB blasts	None	WT1/BIRC5

HCT, hematopoietic cell transplantation; PB, peripheral blood; BM, bone marrow; AML, acute myeloid leukemia; ALL, acute lymphoblastic leukemia; MDS, myelodysplastic syndrome; MRD, minimal residual disease; NED, no evidence of disease; SOC, standard of care; M, male F, female. Shading denotes patients who had evidence of disease at the time of a ddPCR test. In the setting of multiple tests, the highest disease burden was chosen for this test. All TAA/ABL1 that were positive throughout course are listed. **Table 3** shows the time course for SOC and TAA/ABL1 for patients with multiple tests.

In patients with AML, 10/18 (56%) had active leukemia at the time of ddPCR. Of the 10 patients with active AML, all 10 (100%) were positive for at least one TAA, 80% (8/10) were positive for WT1, 40% (4/10) were positive for PRAME, and 60% (6/10) were positive for BIRC5 at any time point tested (**Figure 2E** **).** 60% (6/10) were positive for more than one TAA (**Figure 2E** **).** Of AML patients in clinical remission, 50% (4/8) demonstrated ≥1 TAA, of which two of these four relapsed in the immediate monitoring period, suggesting that the ddPCR may serve as a sensitive biomarker of disease relapse. Of the 13 patients with ALL (B-ALL: n=12; T-ALL: n=1), 9 (69%) had active leukemia at the time of ddPCR assay, though ≥1 TAA were identified in only 7 (54%). Of those with active leukemia, 67% (6/9) were positive for BIRC5, 33% (3/9) for WT1, and 22% (2/9) for PRAME. Thirty-three percent (3/9) were positive for more than 1 TAA (**Figure 2F** **)**.

Combining ALL and AML patients with no evidence of disease at the time of ddPCR evaluation, 58% (7/12) were also negative for all three TAAs (**Table 2** **)**. Active disease status was associated with TAA positivity (1+ TAA vs 0 TAA) with an odds ratio=10.67, (p=0.0070, 95% confidence interval 1.91 – 59.62). The area under the curve is 0.7544.

### Available evaluation of TAA/ABL1 ratios in blood and bone marrow

**Figure 3:**
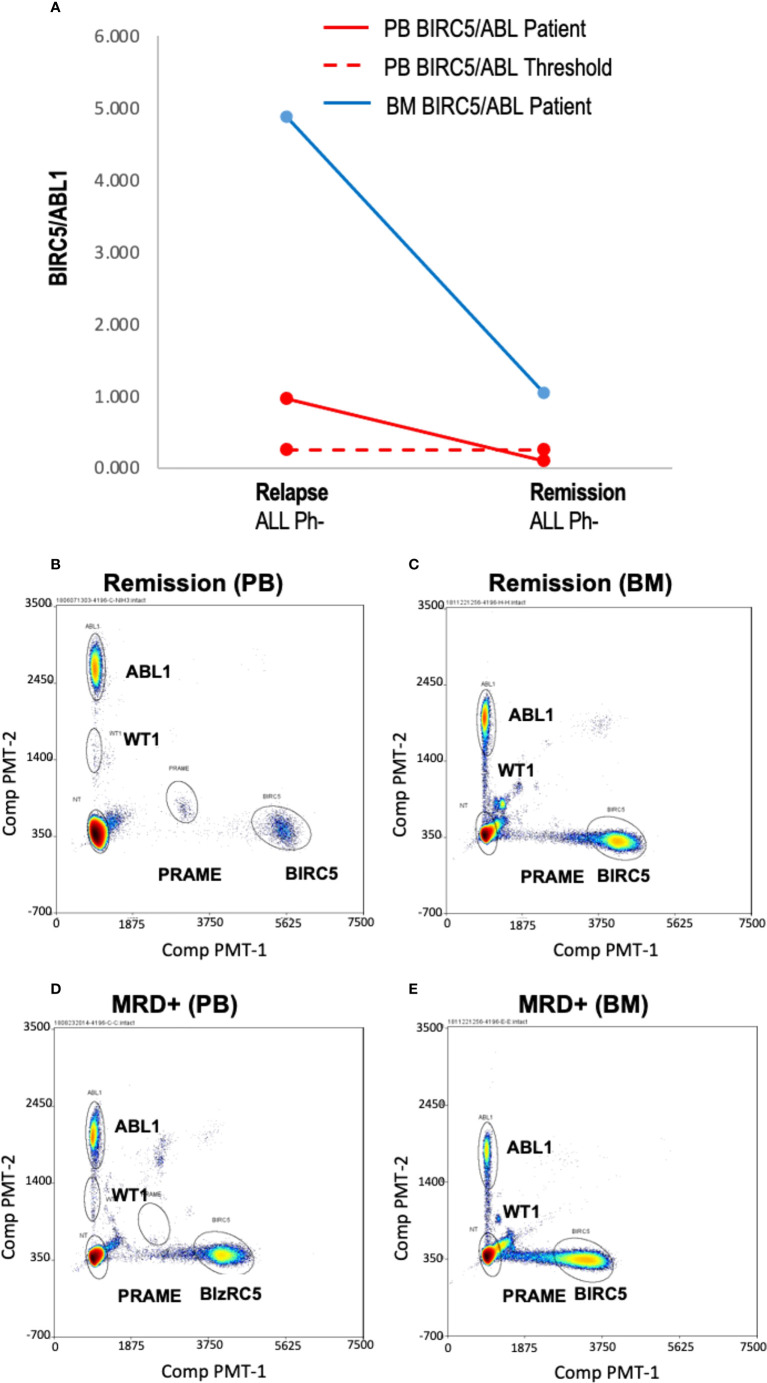
Blood and marrow TAA/ABL1 ratios in a patient with responsive acute leukemia. **(A)** Summary data of BIRC5/ABL1 positive ratios for patient ALL-02 in both blood (grey line) and bone marrow (orange line) during MRD+ active disease (d 21 relapse) and remission (day 100 confirmed by flow cytometry). Dotted line indicates healthy donor blood threshold value. Threshold for BM unknown. **(B–E)** Representative bivariate dot plots from ALL-02 BM **(B)** and PB **(D)** with 2% marrow blasts and during remission (BM: C; PB: E).

Few samples were available to correlate blood TAA/ABL1 ratios and marrow disease burden, (n=4 samples; n=3 patients). If peripheral blood TAA/ABL1 levels are a reliable proxy for disease in the marrow, the marrow test should show a higher TAA/ABL1 ratio compared to the peripheral blood. In one sample (ALL-02), contemporaneous marrow and blood was available. The blood sample was positive for BIRC5/ABL1 and, as expected, the ratio in the marrow was much higher than that of the blood, (**Figures 3A-E**). Assayed at two time points, day 21 and 100 after HCT, the BIRC5/ABL1 ratio declined in the marrow and peripheral blood concurrent with diminished clinical disease burden (MRD+ to negative disease burden by flow cytometry). Two other patients had marrow available for testing (AML-5, ALL-10). One had insufficient peripheral blood cells at the same time point, but had a positive BIRC5/ABL1 ratio at an earlier time point; the other lacked TAA positivity in the blood precluding analysis to correlate with marrow.

### TAA/ABL1 ddPCR detected intra-patient responses in patients and correlated with clinical responses

**Figure 4:**
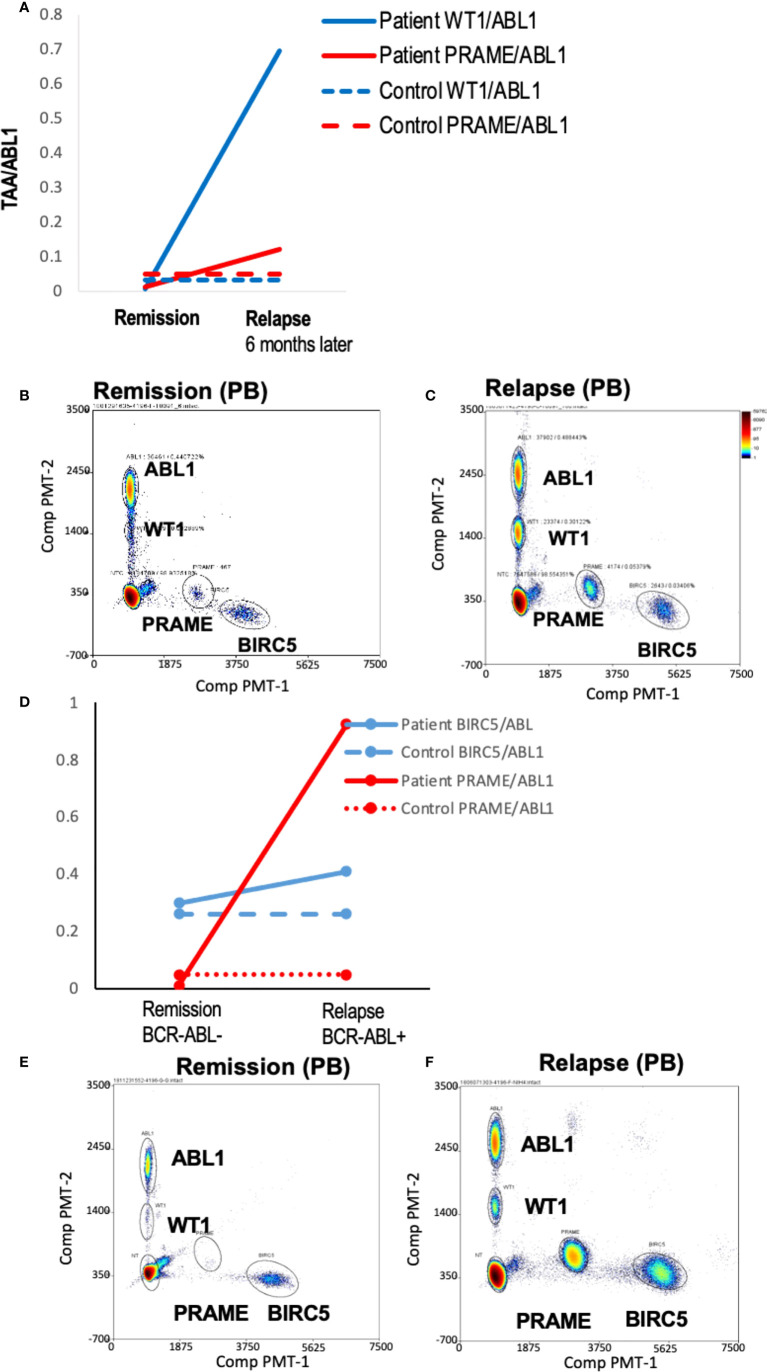
Serial blood TAA/ABL1 ddPCR levels in patients with clinical evidence of acute leukemia. Positive blood TAA/ABL1 ratios graphed over time for patients with clinical evidence of progressive disease **(A)** AML-10 and **(B)** AML-6. Solid lines denote patient blood sample data and dotted lines healthy donor threshold values. Representative raw data bivariate dot plots for graphs A and B show AML-10 in remission **(C)** and relapse **(D)**, and AML-6 in remission **(E)** and relapse **(F)**. AML, acute myeloid leukemia; HCT, hematopoietic cell transplant; PB, peripheral blood; BM, bone marrow; Solid lines = patient ratios. Dotted lines = healthy donor thresholds. Grey: BIRC5. Red: PRAME. Black: WT1. Black open circles: BCR/ABL1 clinical test.

**Figure 5:**
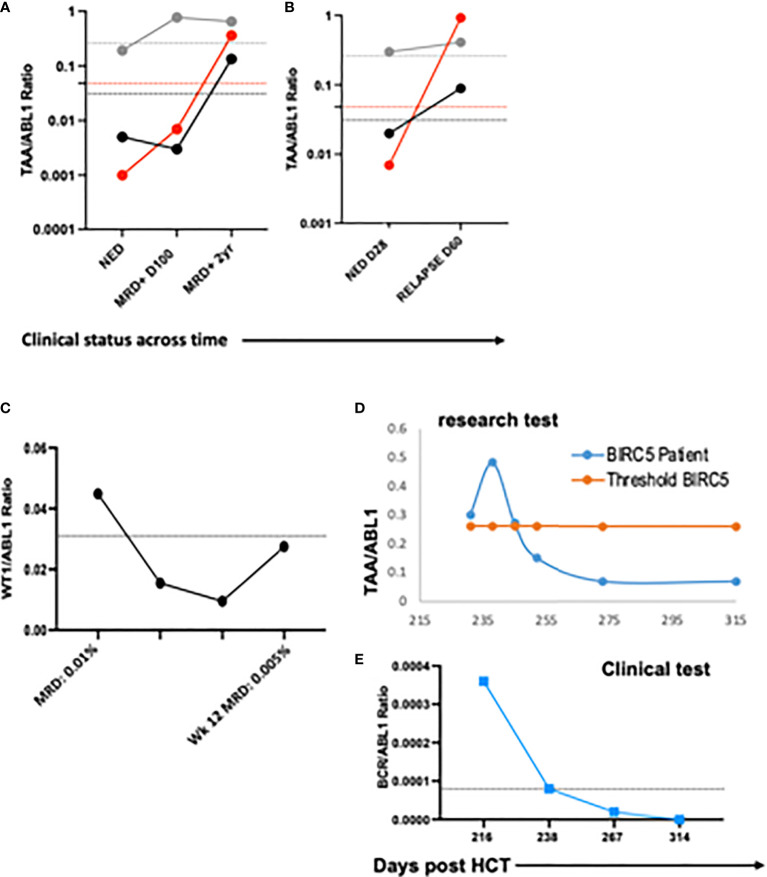
Serial blood TAA/ABL1 ratios and relationship to standard of care tests. The number of TAA/ABL1 ratios above the threshold of normal increased over time with concomitant clinical disease progression in two patients ALL-03 (**(A)** and AML-09 **(B)**. Positive blood TAA-ABL1 ratios graphed over time for two patients with clinical evidence of responsive disease AML-15 **(C)**, and ALL-11 **(D)**. Clinical BCR/ABL ratios for patient ALL-11 **(E)** who experienced disease response. Y axes plotted on log scale to separate data points for easy visualization. Solid lines connect patient blood ratios and dotted lines indicate healthy donor thresholds for BIRC5 (grey), PRAME (red) and WT1 (black). TAA, Tumor-Associated Antigen; MRD+, minimal residual disease positive (by clinical BCR/ABL PCR); NED, no evidence of disease; DZ+, clinical evidence of disease.

To demonstrate the feasibility of the ddPCR assay to serve as a biomarker of disease response post-HCT similar to other MRD tests (e.g. BCR-ABL), serial samples were assayed. All HCT patients with samples tested at multiple time points post-HCT were included (**Table 3**). Of the 16 patients included in the analysis, 12/16 (75%) had concordant clinical and ddPCR results in the timeframe assayed. Half of these concordant patients (n=12) had progressive disease. Two representative patients are shown, one of which was MRD+ (AML-10, AML-06, **Figures 4A-F** **).** Of these concordant progressive disease patients, 2 also showed upregulation of TAAs over time (ALL-3, AML-9, **Figures 5A, B** **)**. Two of these concordant patients had partial or complete response with MRD level disease (AML-15, ALL-11, **Figures 5C, D** **).** One of these two had concurrent serial MRD monitoring of BIRC5/ABL1 ratios permitting comparison with a SOC peripheral blood MRD test and showed correlation between the ddPCR and the BCR-ABL test for surveillance (ALL-11; **Figures 5D, E** **)**. The remaining 4 patients of the 12 concordant patients included 3 continuous remissions (without ddPCR positivity) and one with persistent MRD level of disease and similarly persistent positivity by ddPCR. There were 4 discordant patients, of whom 3 showed greater burden of disease than SOC testing, with the clinical course paralleled in both tests, showing improvement or progression. Finally, one ALL patient had disease without a positive ddPCR value, suggesting that this ALL could not be monitored *via* the ddPCR method.

**Table 3:** Summary of Serial ddPCR TAA/ABL1 levels and correlation with SOC tests .

Patient #	ddPCR + at any point/TAA/ABL1/time difference	Blood ddPCR change	SOC test change	Interpretation
ALL-2	BIRC5 1 month	0.97 (+) ➔ 0.01 (BTP)	MRD+ 2% blasts BM (flow) ➔ NED (BM flow-)	CR by ddPCR and SOC
ALL-3	WT1/PRAME/BIRC5 1 month ➔ 24 months	BTP ➔ 0.78 (+) BIRC5 ➔ (+) WT1 0.13/(+) PRAME 0.36/(+) BIRC5 0.65	CNS+/BM NED BCR-ABL- ➔ BCR-ABL+ ➔relapse/refractory BM/BCR-ABL+	PD by ddPCR and SOC at 1 month and 24 months
AML-6	WT1/PRAME/BIRC5 1 month	(+) 0.3 BIRC5 WT1/PRAME BTT ➔ (+) 0.09 WT1/(+) 0.92 PRAME/0.41 BIRC5	BCR-ABL 0.06% PB, BM flow FISH - ➔ Relapse AML BM 70-80% myeloblasts (flow)	PD by ddPCR and SOC
ALL-8	BIRC5 1.5 months	0.45 (+) ➔ 4.65 (+)	Chloroma mass ➔ stable by Recist	PD by ddPCR, SD by SOC then progression (post testing)
AML-8	BIRC5 1 month	BTP ➔ (+) 0.81	NED ➔ MRD+ BM 2% blasts by flow	PD by ddPCR and SOC
AML-9	WT1, BIRC5 1 month	(+) BIRC5 0.81/BTP WT1 ➔ (+) BIRC5 0.38 (+) WT1 0.88	NED by PB ➔ relapse “replaced” blasts morph	PD by ddPCR and SOC
AML-10	WT1/PRAME/BIRC5 6 months ➔ 1 month ➔ 1 month	BTP ➔ (+) WT1 0.7 (+) PRAME 0.12 ➔ (+) WT1 0.07 (-) PRAME 0.02 (+) BIRC5 0.75	NED by BM ➔relapse (PB blasts morph)➔ CR MRD+ ➔ relapse AML	PD by ddPCR and SOC ➔ CR MRD+ by SOC and ddPCR/new BIRC5 clone/loss of PRAME
AML-11	WT1/BIRC5 3 weeks	(+) WT1 0.06 ➔ (+) Wt1 0.3 (+) BIRC5 0.3	NED by PB (no BM > 2 month)➔ relapse PB blasts morph	PD by ddPCR and SOC
AML-12	BIRC5 5 months ➔ 10 months	(+) BIRC5 0.46 ➔ (-) BIRC5 0.13 ➔ no ddPCR at relapse	NED but no BM/low platelets ➔ platelets recover ➔ relapse chloroma	CCR by SOC and CR ddPCR
ALL-11	BIRC5/WT1 3 weeks➔ 3 months	(+) BIRC5 0.3 ➔ (+) BIRC5 0.15 ➔ (+) WT1 0.37	BCR-ABL+ ➔ BCR-ABL - ➔ BCR-ABL+	MRD+ by SOC and ddPCR
AML-13	BIRC5/WT1 1.5 months	(+) BIRC5 0.32 ➔ (+) WT1 0.04	NED (on vidaza)	MRD+ by ddPCR CR by SOC
AML-14	None	——	NED	CCR by ddPCR and SOC. Relapse BM morph +4 months- no ddPCR in prior 3 months
AML-15	WT1 3 months	(+) WT1 0.045➔ BTP	MRD+ MLL PCR BM 0.01% ➔ MRD+ MLL PCR BM 0.005%	PR by ddPCR and SOC
AML-16	None	—–	NED	CCR by ddPCR and SOC
ALL-4	None	——	None	CCR by SOC and by ddPCR
ALL-7	None	——	Relapse PB blasts by flow ➔ response	CR by SOC but CCR by ddPCR

HCT, hematopoietic cell transplantation; PB, peripheral blood; BM, bone marrow; AML, acute myeloid leukemia; ALL, acute lymphoblastic leukemia; BTP, below the threshold of positivity (WT1/ABL1, 0.031; PRAME/ABL1, 0.048; BIRC5/ABL1, 0.26); MRD, minimal residual disease; NED, no evidence of disease; SOC, standard of care; CCR, continuous complete remission; PR, partial response; CR, complete remission; PD, progressive disease; flow, flow cytometry method. Shading denotes patients who had matching ddPCR and SOC tests. All values not listed (e.g. of TAA/ABL1 ratios were BTP.

### TAA/ABL1 levels and survival

**Figure 6:**
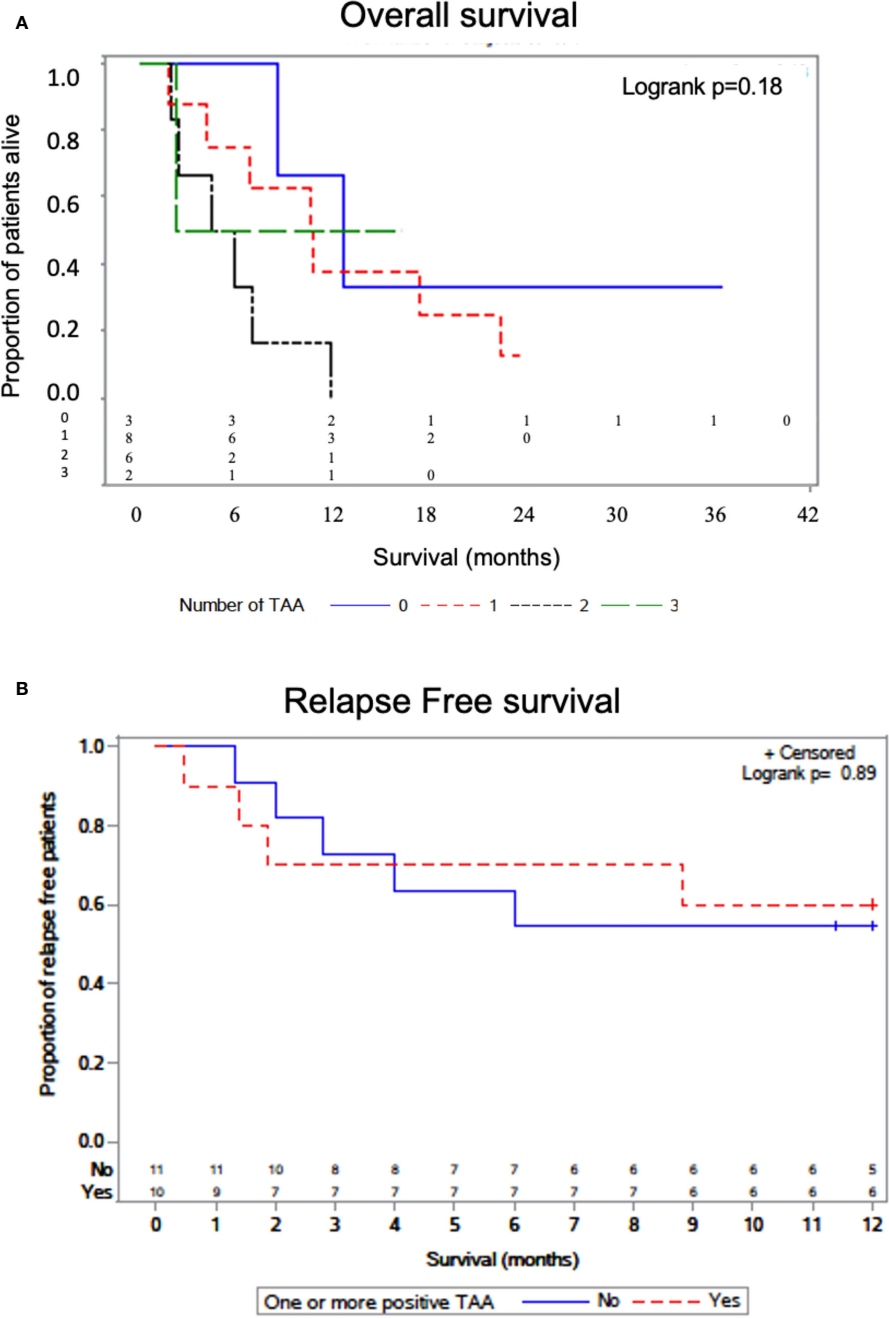
Overall survival by number of TAAs detected by ddPCR. Kaplan-Meier curve with log-rank test showing survival of patients as a function of the number of TAA detected in active leukemia *via* TAA/ABL1 ratios **(A)**. Relapse free survival curve as a function of the one or more TAA detected in active leukemia *via* TAA/ABL1 ratios **(B)**. Number at risk below the graph grouped as per the number with each TAA (0,1,2 and 3).

We interrogated whether increased numbers of TAA/ABL1 correlated with survival. Median survival was lower for patients with one or two TAAs as compared to those without (, but the difference was not statistically significant P=0.176). In the small number of patients with 3 TAAs, one received CAR-T cells for relapsed B-cell ALL after HCT, achieving durable survival and exceeding that of the other survival curves (**Figure 6A** **)**. We also assayed time to relapse. There was no statistical difference in time to relapse comparing those with and without a positive TAA/ABL (**Figure 6B**).

## Discussion

The ability to sample from blood, rather than the bone marrow, would represent a less-invasive and thus more frequently obtainable method of surveillance for minimal/measurable residual leukemia disease and disease progression after HCT. We therefore developed a multiplex ddPCR to simultaneously and sensitively detect three tumor associated antigens commonly expressed in relapsed/refractory acute myeloid and lymphoid leukemias. We confirmed the test measured the TAA by showing that ddPCR detected TAA identified by flow cytometry. Then, we showed that the TAA/ABL1 levels from patients with MRD+ or relapsed acute leukemias were significantly higher than those of healthy control individuals. While qPCR could be used to measure individual blood TAAs, the quantity of mRNA required limits this approach for testing ≥1 TAA in relapsed patients with leukopenia. Given that many leukemias expressed only one TAA, this multiplex test approach was valuable for broader surveillance of tumor burden *via* the peripheral blood in our multiply relapsed cohort.

As expected, most healthy individuals had low blood levels of WT1, PRAME, and BIRC5 (survivin), except for one donor whose levels were anomalously high. This may have reflected undiagnosed cancer or pregnancy, as the TAA are fetal antigens, though limited information about healthy donors precluded interpretation. The median levels of the remaining included donors were similar to previously published qPCR data for WT1 and PRAME in the peripheral blood of healthy subjects (46, 56). Further, the highest level of each TAA in the healthy donors was chosen as a stringent ‘normal’ threshold for controls. Our data was highly reproducible using just 3 million cells, a factor of critical importance as patients with relapsed acute leukemia often experience leukopenia. More patients would be needed to define the ‘true’ threshold of normal (and those for whom false positives would be likely). In addition, the full characteristics of the ddPCR test will need to be fully characterized, including the level of detection, the level of blank, the positive and negative predictive values in future studies. That said, many such MRD monitoring tests rely upon serial increasing values to identify advancing leukemia (e.g. immunoglobulin receptor sequencing or BCR-ABL levels), thus diminishing the emphasis of certainty regarding the impact of a single value that is ‘positive’ at a single timepoint post-HCT.

As expected, the mRNA ratio of the TAAs was higher in the peripheral blood of patients with concurrent evidence of acute leukemia as compared to the healthy donors, consistent with detection of leukemia. While sample availability limited testing marrow and blood simultaneously to a single patient at two timepoints, this patient confirmed the same tumor associated antigen in the marrow as the blood, at much higher levels, indicating our blood test reflected marrow disease burden in this patient. Further, the response in the marrow mirrored that of the peripheral blood in this single patient who had both contemporaneous samples for testing and positive TAA/ABL1 in the peripheral blood. While the marrow level remained higher than blood after the remission timepoint, given that survivin (BIRC5) is present in healthy hematopoietic stem cells, this may reflect a normal level in the setting of marrow repopulation, though further studies would be needed to better evaluate this.

The ddPCR multiplex test responses reflected the clinical responses in patients with serial samples tested, concordant in 75% and accurately denoting positive or negative disease burden in 15/16 (95%). This included patients who had only MRD level of leukemia burden, suggesting that blood ddPCR sensitivity may detect early progression. Further, our novel ddPCR test mirrored surveillance monitoring of the one patient who had a leukemia evaluable by blood PCR using BCR-ABL. This standard of care clinical test measures blood levels of BCR-ABL/ABL1. The BIRC5/ABL1 levels showed similar reduction of disease burden and a similar time point of crossing a healthy threshold. While more paired samples are needed to demonstrate accuracy as a leukemia surveillance tool, our ddPCR test showed sensitive changes in TAA/ABL1 ratios that matched clinical course even in low level leukemia disease over the course of treatment. Further, while the test was discordant in 25%, 3 of these 4 patients were discordant because the ddPCR test showed higher burden of disease than SOC, as might be expected in a test with higher sensitivity than SOC.

In multiply relapsed/refractory patients after HCT, we showed that most patients with active disease could be detected in the peripheral blood using ddPCR of blood mRNA TAA/ABL1 ratios. The odds ratio of having acute leukemia with a TAA detected by ddPCR was significantly higher than those patients without an elevated TAA, supporting the hypothesis that the ddPCR test would be valuable for surveillance of residual or recurrent acute leukemia. While these TAA have been shown to be expressed in AML and ALL, the reported expression rates vary widely. This likely reflects the inclusion of low risk patients and the fact that most studies focused on PRAME and WT1 for AML and BIRC5 expression for ALL (31, 33, 37, 38, 45–52, 57). Because these TAA are increased in relapsed refractory acute leukemia, and this is the patient population who relapses after HCT, it is not surprising that our cohort was enriched for TAA+ leukemias. We detected at least one TAA in the peripheral blood of 84% of patients after HCT with evidence of acute leukemia, in 100% of AML samples and 67% of ALL. Of our HCT cohort, only 32% (10/31) of patients could be monitored by the standard of care measurement of BCR-ABL/ABL1 in the peripheral blood. It is estimated that only 2-4% of pediatric and less than 30% of adult acute leukemia patients would have this Philadelphia chromosome mutation permitting disease detection in the peripheral blood (58). Given that bone marrow tests are performed infrequently, typically 60 and 100 days after HCT, peripheral blood monitoring could greatly enhance MRD detection. Using marrow studies to monitor acute leukemia, most of the B lymphoid acute leukemias would be likely be detected at a low level by next generation sequencing; however, only 50% (9/18) of AMLs in our cohort had a molecular marker to identify MRD. Thus, other methods may serve as better biomarkers for relapse of B-ALL. For AML patients with active disease, combining this novel ddPCR TAA assay and the standard of care molecular markers for MRD, all AMLs could be detected at the level of PCR in our cohort (9, 59, 60). All of these AML patients were positive for at least 1 TAA by ddPCR, permitting sensitive, serial, real-time detection of low level disease burden in the peripheral blood that was correlated with response. Thus, our approach could provide an option for AML patients post-HCT that is underserved by current MRD monitoring. For patients with AML undergoing HCT, this is a critical need, as there are few treatment options and these are most successful at low level disease burden, e.g. donor lymphocyte infusions (1).

Our ddPCR method of surveying acute leukemia burden by blood TAA/ABL1 may be valuable for other tumors that also express these TAAs, such as chronic leukemias, lymphomas, breast, lung, pancreas and colon cancer. (41, 61–64). In addition, our ddPCR test could be useful for monitoring response or changes in response to immunotherapies that target these TAA, e.g. WT1 vaccine, T cells expanded using pooled peptides of WT1, Survivin, and PRAME; HLA-A*0201 restricted WT1-specific or PRAME-specific modified CD8 T cells; or genetically modified HLA-A*24:02 restricted WT1 (65–69). Real-time detection of these TAA mRNA could provide a sensitive assessment of response, and detect disease burden changes between invasive marrow tests. It could also direct the timing of cellular therapy administration if the TAA mRNA level first declined and then rose rapidly between doses. Alternatively, these data could inform changes of tumor TAA expression, directing alternate approaches as TAA levels change during therapy. Several of our patients who progressed demonstrated an increased number of TAA antigens number over time. This was not unexpected since these TAA encode for anti-apoptotic proteins, which would confer a leukemia survival advantage (32, 33, 36, 37, 44, 51, 57, 70).

Limitations of this work included limited leukemia and healthy control sample numbers, though our data are in line with those of other mRNA publications measured from the peripheral blood. Another limitation is the lack of information on the differing mutational profiles of the patient tumors due to lack of availability of samples. In addition, lack of availability of marrow specimens limited the ability to test contemporaneous blood samples and to generate a healthy donor marrow threshold or perform multiple experiments comparing contemporaneous blood and marrow TAA/ABL1. However, the correlation with clinically identified disease status, the concordance of TAA/ABL1 in the marrow and blood of the one evaluable patient, and confirmation of the ddPCR-identified TAAs by flow cytometry in a leukemia cell line, all support the hypothesis that our test measured leukemia burden after HCT and may serve as a biomarker of relapse after HCT. Many patients (5/12; 42%) had elevated TAA/ABL1 ratios without evidence of active disease by standard of care and this could potentially be interpreted as a false positive result. However, 40% (2/5) of these ultimately relapsed during study evaluation, which could suggest that our test was more sensitive than standard of care tests rather than falsely positive. It is unknown if the remaining three patients subsequently relapsed after surveillance. An additional consideration is that the ddPCR test could also yield a false negative test, either from lack of TAA expression within the leukemia, or a mutation within the gene target, as has been reported with WT1 in certain acute leukemias (71). In addition, the location of the WT1 droplets in the multiplex test could lead to false positives, though this also affects the healthy controls values, thus our approach to include only those that exceeded healthy control levels should mitigate this risk. The survival analysis is limited by both few patient numbers in each of the TAA/ABL1 cohorts and heterogeneity in terms of time assayed post-HCT. Finally, these data represent a preliminary study and as such validations in larger cohorts are needed to evaluate the positive and negative predictive value of this test and the test performance characteristics.

In summary, we have devised and tested a minimal/measurable residual disease test to survey acute leukemia using multiplex ddPCR, which could be valuable as a biomarker of AML assessment of response, prognosis, and disease stratification. If these results are validated in larger cohorts, it could add to the emerging methods for AML MRD detection, and ultimately improve outcomes for relapsed refractory acute myeloid leukemias after HCT.

## Data availability statement

The original contributions presented in the study are included in the article/**Supplementary Material**. Further inquiries can be directed to the corresponding author.

## Ethics statement

The studies involving human participants were reviewed and approved by Children’s National Institutional Review Board and National Institutes of Health Institutional Review Board. Written informed consent to participate in this study was provided by the participants’ legal guardian/next of kin.

## Author contributions

KW, MS, MG, JN developed the method. MS generated the primary data. Data analysis was performed by MS, KW, MG, JHC MG, MS, and KW wrote the manuscript. Statistical analysis was performed by SV RG, CK, SK, EP, KP assisted with sample procurement, data interpretation, and edited the manuscript. All authors contributed to the article and approved the submitted version. KW wrote the manuscript with assistance from MG/MS.

## Funding

This research was supported by a Leukemia Lymphoma Society Translational Research Program grant 6562-19, a grant from the Rising Tide Foundation, Ben's Run Gift/Ben's Run Inc. Research reported in this publication was supported in part by the National Cancer Institute Cancer Center Support Grant P30CA225520 awarded to the University of Oklahoma Stephenson Cancer Center and used the Biostatistics and Research Design Shared Resource. The content is solely the responsibility of the authors and does not necessarily represent the official views of the National Institutes of Health. Research reported in this publication was supported in part by the Oklahoma Tobacco Settlement Endowment Trust awarded to the University of Oklahoma//Stephenson Cancer Center. The content is solely the responsibility of the authors and does not necessarily represent the official views of the Oklahoma Tobacco Settlement Endowment Trust.

## Conflict of interest

The authors declare that the research was conducted in the absence of any commercial or financial relationships that could be construed as a potential conflict of interest.

## Publisher's note

All claims expressed in this article are solely those of the authors and do not necessarily represent those of their affiliated organizations, or those of the publisher, the editors and the reviewers. Any product that may be evaluated in this article, or claim that may be made by its manufacturer, is not guaranteed or endorsed by the publisher.
