# Supplementary material for: Peripheral blood marker of residual acute leukemia after hematopoietic cell transplantation using multi-plex digital droplet PCR
Source: Front Immunol. 2022 Sep 29;13:999298. doi: 10.3389/fimmu.2022.999298 (PMC9556966; doi:10.3389/fimmu.2022.999298)
Supplement: Supplementary file 1 [file DataSheet_1.pdf]

Supplementary Table 1

	WT1/ABL1	PRAME/ABL1	BIRC5/ABL1
BC100114-1	0.02	1.88	0.81
BC100114-2	0.02	2.14	0.84
BC100114-3	0.02	1.9	0.79
BC100114-4	0.02	1.96	0.82
BC100114-5	0.018	1.56	0.64
BC100114-6	0.018	1.65	0.63
BC100114-7	0.02	1.87	0.67
BC091417-1	0.02	1.76	0.75
BC091417-2	0.02	2.03	0.58
BC091417-3	0.016	1.31	0.61
BC091417-4	0.016	1.25	0.6
BC091417-5	0.017	1.3	0.6
BC091417-6	0.016	1.25	0.62
BC091417-7	0.016	1.34	0.72

Supplementary Table 2: Healthy Control Ratios of TAA/ABL1

Sample#	WT1/ABL1	PRAME/ABL1	BIRC5/ABL1
1	0.006	0.003	0.052
2	0.0053	0.0013	0.0462
3	0.0064	0.0013	0.17
4	0.007	**0.048**	0.06
5	0.008	0.039	0.071
6	0.0061	0.0239	0.0815
7	0.0111	0.044	0.03
8	**0.0309**	0.037	0.03
9	0.0055	0.0008	0.0146
10	0.0025	0.025	0.05
11	0.0025	0.002	0.01
12	0.002	0.000	0.036
13	0.0026	0.0005	0.0216
14	0.0024	0.000	0.01
15	0.002	0.002	**0.26**
16	0.002	0.0008	0.0448
17	0.0026	0.002	0.04
18	0.0019	0.001	0.05
19	0.036	1.68	0.16
20	0.002	0.001	0.05
**THRESHOLD**	**0.031**	**0.048**	**0.26**
**THP-1 CELLS**	**0.657**	**1.383**	**3.105**

Table legend: Healthy control TAA/ABL1 are listed. The threshold of positive is the highest value of the remaining healthy controls for each TAA/ABL1 (in bold) after excluding the outlier (examined in comparison to published ratios).
